# Sucralose and the Gut–Immune Axis: Emerging Evidence Linking Dysbiosis, Barrier Alterations, and Implications for Colitis and Colorectal Cancer Immunotherapy

**DOI:** 10.3390/biomedicines14040917

**Published:** 2026-04-17

**Authors:** Aranza Mejía-Muñoz, Jessica Cedillo Monter, Héctor Iván Saldívar-Cerón, Galileo Escobedo, Sonia Leon-Cabrera

**Affiliations:** 1 Unidad de Biomedicina (UBIMED), Facultad de Estudios Superiores-Iztacala, Universidad Nacional Autónoma de México, Tlalnepantla 54090, Mexico; 2 Carrera de Médico Cirujano, Facultad de Estudios Superiores-Iztacala, Universidad Nacional Autónoma de México, Tlalnepantla 54090, Mexico; ivansaldi@iztacala.unam.mx; 3 Laboratorio de Inmunometabolismo, División de Investigación, Hospital General de México Dr. Eduardo Liceaga, Ciudad de México 06720, Mexico; gescobedog@msn.com

**Keywords:** sucralose, gut, immunotherapy, colorectal cancer, dysbiosis, immune exhaustion, immune checkpoint inhibitors

## Abstract

Sucralose is one of the most widely used non-nutritive sweeteners and has long been considered metabolically inert and safe within established acceptable daily intake levels. However, emerging evidence suggests that chronic exposure to sucralose may alter gut microbial composition, epithelial barrier function, mucosal inflammation, and immune responses. This review examines current experimental and clinical evidence on the effects of sucralose on the gut–immune axis, with particular attention to its potential implications for colitis and colorectal cancer (CRC). Preclinical studies indicate that sucralose may reduce beneficial short-chain fatty acid-producing taxa, alter microbial metabolic pathways, disrupt epithelial barrier-related molecules, and promote inflammatory and immune changes associated with colitis severity and inflammation-driven tumorigenesis. Experimental evidence also suggests that sucralose may impair CD8^+^ T-cell fitness and reduce responsiveness to immune checkpoint inhibitors through microbiome-dependent mechanisms involving altered arginine and citrulline metabolism. Human studies further indicate that sucralose can modify gut and oral microbiome composition and influence metabolic responses, although these effects appear heterogeneous and context-dependent. Overall, the current literature suggests that sucralose may act as a modifier of microbiome–immune interactions in susceptible settings, but most mechanistic evidence remains preclinical, and human data are still insufficient to establish causality. These findings highlight the need for prospective studies to determine whether sucralose-associated microbial and immune alterations translate into clinically meaningful effects in colitis, CRC, and immunotherapy response.

## 1. Introduction

Sucralose is a common food additive that sweetens foods and drinks without adding calories [1,2]. As a non-caloric sweetener, it is among the most widely used. It is found in over 4500 commercial products and is a key ingredient in the food industry [2,3]. Discovered in 1976, sucralose is chemically 1,6-dichloro-1,6-dideoxy-β-D-fructofuranosyl-4-chloro-4-deoxy-α-D-galactopyranoside, an artificial chlorinated sucrose derivative [1,4,5]. Its popularity stems from unique physicochemical properties: high solid-state stability, resistance to degradation over a wide pH range, and sweetness about 600 times that of sucrose [1].

For decades, sucralose was considered inert and safe. In 1998, the Joint FAO/WHO Expert Committee on Food Additives labeled it non-toxic. The U.S. Food and Drug Administration (FDA) and European Food Safety Authority (EFSA) set acceptable daily intake (ADI) at 5 mg/kg/day and 15 mg/kg/day, respectively [6,7]. Sucralose, initially promoted as a healthier sugar alternative for obesity and metabolic disorders, now faces scrutiny. Recent reports link its use to systemic inflammation, microbiome disruption, and metabolic dysregulation [8].

Emerging evidence suggests that sucralose consumption may affect gut physiology beyond its intended metabolic neutrality by influencing epithelial integrity, microbial composition, and host immune responses. Recent preclinical studies indicate that chronic exposure to sucralose may alter the gut microbiome and immune pathways relevant to tumor progression and antitumor immunity. In experimental systems, these alterations have been associated with changes in the tumor microenvironment, T-cell dysfunction, immune exhaustion, and reduced responsiveness to cancer immunotherapy. Rather than reexamining previously described compositional changes in the microbiota, which have been reviewed elsewhere [9,10], this article focuses on integrating mechanistic evidence linking sucralose-induced dysbiosis, barrier dysfunction, and immune modulation with inflammation-driven carcinogenesis and impaired antitumor immunity.

To this end, we first summarize the effects of sucralose on gut microbiota composition and function, intestinal barrier integrity, and mucosal inflammation. We then examine its immunomodulatory effects on T-cell responses and their potential implications for colon tumor development and immunotherapy efficacy. Finally, we discuss the clinical and translational relevance of chronic sucralose consumption and emphasize the need for further studies to determine whether these findings translate into clinically meaningful effects on colorectal cancer susceptibility and treatment outcomes.

## 2. Methods

This manuscript was designed as a narrative review informed by a structured literature search. The relevant literature was identified through searches of PubMed/MEDLINE and Scopus. The last search was conducted in February 2026. Search terms were combined using Boolean operators and included: “sucralose,” “non-nutritive sweeteners,” “artificial sweeteners,” “Splenda,” “gut microbiome,” “dysbiosis,” “intestinal barrier,” “mucosal immunity,” “inflammation,” “colitis,” “colorectal cancer,” “Fusobacterium,” “short-chain fatty acids,” “arginine,” “citrulline,” “immunotherapy”, and “immune checkpoint inhibitors/PD-1.” Original research articles and high-quality reviews published in English between 2007 and 2026 were considered. Priority was given to studies with clearly defined exposures, appropriate controls, and relevance to microbiome–immune–tumor interactions. Human studies were prioritized when available. Preclinical studies were included when they provided mechanistic insights not feasible in humans or addressed clinically relevant models, such as DSS-induced colitis or colitis-associated colorectal cancer. In addition, reference lists of key articles were hand-searched to identify further relevant studies. Given the heterogeneity in exposure assessment, model systems, and outcome measures across the available literature, this review does not aim to provide a systematic quantitative synthesis. Instead, it focuses on integrating convergent findings, identifying mechanistic themes, and highlighting areas of inconsistency or limited evidence. Overall, the review considered 35 studies: 17 in experimental animal models; 9 in human studies (Clinical/Trials); 5 in mixed studies (translational); and 4 in vitro/basic science (Appendix A).

To translate sucralose doses from animal experiments into approximate human-equivalent exposures, we calculated the human-equivalent dose (HED) using the Body Surface Area (BSA) normalization method. This follows FDA Industry Guidance (2005) [11]. To standardize sucralose exposure across included clinical trials, daily sucralose intake was converted to a body weight-adjusted dose (mg/kg/day). For studies reporting absolute daily consumption (such as mg/day or packets/day), the estimated daily intake (EDI) was calculated using the following formula:

EDI (mg/kg/day) = ((Total daily sucralose intake (mg/day))/(Body Weight (60 kg)))

In accordance with international nutritional and pharmacological profiling standards [7], a reference body weight of 60 kg was used for all calculations to ensure consistency across the dataset, aligning with the standard weight used in FDA dose-scaling guidelines [11]. This standardized dose was then used to compare the intervention levels with the ADI of 5 mg/kg/day established by regulatory agencies [6].

## 3. Sucralose Alters Gut Microbiome Composition and Function

Previous studies in both human and experimental models have extensively documented the effects of sucralose on gut microbiota composition and function [10,12,13]. The aim of this review is to integrate these findings and examine how sucralose-induced alterations in microbial and immunological systems may influence tumor-promoting pathways and, in turn, compromise the efficacy of cancer immunotherapy.

Across multiple CRC patient cohorts, the gut microbiota exhibits reproducible, disease-associated microbial signatures, reinforcing dysbiosis as a hallmark of colorectal carcinogenesis [14,15,16]. Recent advances indicate that the gut microbiota not only contributes to CRC development but also represents a promising non-invasive diagnostic biomarker. Using a large integrated dataset, Tričković et al. [17] constructed a comprehensive catalog of human gut microbiota subspecies, defined as operational subspecies units (OSUs), and demonstrated that subspecies-level microbial signatures outperform species-level profiles in predicting CRC from stool samples. By applying machine learning to OSU-based datasets, the authors detected approximately 90% of CRC cases, a performance close to that of colonoscopy and superior to current non-invasive screening methods, thereby highlighting the diagnostic potential of microbiota-derived signatures for early CRC detection. These findings underscore that even subtle shifts in microbial composition or metabolic function may have clinically meaningful implications for CRC risk stratification.

Classic features of CRC-related dysbiosis include reductions in Firmicutes and increases in Bacteroidetes, together with the expansion of pathogenic species such as *Escherichia coli* (pks+), *enterotoxigenic Bacteroides fragilis* (ETBF), and *Fusobacterium nucleatum* [18,19]. Butyrate-producing taxa from the Lachnospiraceae family, including *Roseburia* and *Eubacterium rectale*, are consistently depleted in CRC. In vivo, butyrate generation primarily depends on clostridial cluster IV (*Faecalibacterium prausnitzii*) and cluster XIVa (*E. rectale, Roseburia*) [4]. The loss of short-chain fatty acid (SCFA)-producing bacteria reduces colonic butyrate availability, promoting oxidative DNA damage, impairing apoptosis of genomically stressed cells, facilitating tumor cell expansion, and enhancing pro-carcinogenic microbial enzyme activity [20,21].

Butyrogenic microbes are key regulators of intestinal homeostasis. Colonocytes rely on butyrate as their main energy source, and butyrate oxidation enhances epithelial oxygen consumption, thereby helping to maintain a hypoxic mucosal environment. This physiological hypoxia stabilizes the hypoxia-inducible factor (HIF), which is crucial for preserving barrier integrity and maintaining luminal anaerobiosis. Through these mechanisms, butyrate limits the expansion of facultative anaerobic bacteria and supports epithelial homeostasis [12]. In addition, butyrate strengthens tight junctions by regulating Claudin-1 and synaptopodin, suppresses pro-inflammatory cytokines such as IL-6 and IL-12, and inhibits oncogenic pathways including Akt/ERK, Wnt, and TGF-β [4,22,23]. SCFAs produced by butyrate-producing bacteria also help shape the microbial ecosystem by promoting the production of antimicrobial molecules such as cathelicidins, reuterin, and β-defensin-1, while supporting IgA responses and other anti-inflammatory metabolites [4,24]. Furthermore, species such as *Roseburia* may produce anti-carcinogenic compounds, including shikimic acid and precursors of conjugated linoleic acid [25].

Dysbiosis may also enhance tumor susceptibility by promoting chronic immune activation, leading to T-cell dysfunction in the tumor microenvironment. In colitis-associated cancer (CAC) models, dysbiotic microbiota promotes pro-inflammatory cytokine release and epithelial barrier injury, thereby contributing to carcinogenesis through maladaptive immune activation [26]. Notably, as tumors develop, there is a marked reduction in intratumoral CD8^+^IFN-γ^+^ T-cell infiltration accompanied by increased expression of exhaustion markers, reflecting a transition from early immune hyperactivation to later immune dysfunction. These findings suggest that, in a dysbiotic microbiome, sustained immune activation may accelerate tissue damage and promote T-cell exhaustion [26].

In this context, dietary exposures that alter the microbiota, such as sucralose, may be especially relevant. Emerging evidence from preclinical models (Table 1) suggests that sucralose-induced dysbiosis may reproduce immunological disturbances similar to those observed in colitis-associated tumorigenesis. Chronic sucralose consumption reduces beneficial butyrate-producing taxa, including *Faecalibacterium*, *Roseburia*, and *Ruminococcus*, while enriching pro-inflammatory taxa such as *Bacteroides*, *Fusobacterium*, and Enterobacteriaceae [27,28,29,30,31,32]. The resulting SCFA deficiency may restrict T-cell metabolic fitness, memory formation, and IFN-γ production. At the same time, enrichment of Enterobacteriaceae, *F. nucleatum*, and *Escherichia/Shigella* may activate TLR–NF-κB pathways and drive mucosal cytokine release, thereby promoting macrophage and neutrophil recruitment and reinforcing chronic epithelial stress [33]. However, this relationship should be interpreted as a hypothesis-generating framework rather than definitive evidence of causality. Although sucralose-induced changes in microbial structure and metabolic activity could potentially affect CRC-associated microbial signatures, their significance for CRC remains uncertain. In particular, higher taxonomic resolution at the species and subspecies levels will be necessary before drawing meaningful conclusions regarding CRC-associated microbial patterns or the performance of microbiome-based CRC detection tools.

Human studies further support the effects of sucralose on the gut microbiome. In a randomized controlled trial involving 120 healthy adults who had never previously consumed non-nutritive sweeteners (NNSs), two weeks of sucralose intake at doses below the acceptable daily intake (ADI) induced significant shifts in gut microbiota structure at both the genus and species levels [43]. These changes were mechanistically linked to impaired glycemic responses and involved metabolic pathways, including purine metabolism, which directly influences microbial replication and energy balance. In the same study, sucralose consumption also altered the oral microbiome, including the relative abundance of six Streptococcus species [43]. Consistent with the idea that sucralose may affect microbial communities beyond the gut, experimental studies have shown that it inhibits the growth of oral biofilm-associated bacteria, such as *Porphyromonas gingivalis*, *Streptococcus mutans*, and *Streptococcus sanguinis*, species associated with periodontal disease and cariogenic biofilms [44,45]. Sucralose has also been reported to modulate key metabolic enzymes in *Escherichia coli*, altering propanoate, phosphonate, phosphinate, fatty acid, and pentose phosphate pathways, as well as the biosynthesis of lysine and aromatic amino acids [46]. In addition, in vitro fermentation studies using fecal samples from healthy donors and patients with IBD demonstrated a significant reduction in *Clostridium leptum* (cluster IV) in IBD-derived microbiota, together with altered SCFA profiles under sucralose exposure [30].

Sucralose is absorbed only to a limited extent, meaning that a substantial proportion reaches the colon and is excreted unchanged in feces [5]. However, not all ingested sucralose follows this route, and the metabolic fate of the remaining fraction remains poorly defined [1]. Sucralose-related metabolites of unknown function have been detected in feces and adipose tissue, suggesting that additional processing may occur in at least some individuals [5,47]. Notably, reported inter-subject variability in fecal excretion may partly explain the heterogeneous metabolic responses associated with sucralose exposure.

Emerging evidence suggests that the gut microbiota can influence disease pathophysiology through metabolite-driven interactions with the host. Alterations in microbial composition are frequently accompanied by distinct fecal, serum, and tissue metabolomic profiles, underscoring the systemic consequences of microbiota-induced metabolic remodeling. In particular, microbial pathways involved in lipid metabolism, including fatty acid and glycerophospholipid metabolism, may shape inflammatory signaling and thereby influence disease susceptibility and progression [48]. In addition, non-viable microbial cells or their components, known as paraprobiotics, are increasingly recognized for their capacity to modulate immune responses, enhance mucosal defense mechanisms, and reinforce intestinal barrier integrity by influencing signaling cascades such as NF-κB, MAPKs, and inflammasome pathways [48]. Together, these observations support the concept that microbiota-derived metabolites and microbial components act not only as local regulators of intestinal homeostasis but also as broader modulators of host immune and inflammatory responses. More broadly, shifts in specific bacterial groups may remodel the intestinal microenvironment, favoring the expansion of opportunistic pathogens. By altering local metabolic networks and immune homeostasis, dysbiosis can create permissive conditions for the proliferation of pathogenic species such as *Clostridioides difficile*, further illustrating how microbiota disruption may influence disease susceptibility.

In murine models, chronic sucralose consumption consistently alters the abundance of *Akkermansia muciniphila*, a species notably reduced in CRC patients and recognized for its antitumor effects [49]. Mechanistically, *A. muciniphila* suppresses tumorigenesis by promoting M1-like macrophage activation through the TLR2–NF-κB–NLRP3 axis, a pathway essential for its antitumor efficacy [50]. In parallel, it inhibits the tryptophan–AhR–β-catenin signaling pathway, thereby reducing proliferation and tumor progression in both chemical and genetic CRC models [51]. In clinical studies, depletion of *A. muciniphila* has been associated with gut dysbiosis, increased tumor burden, and reduced survival in CRC patients, underscoring its relevance as both a therapeutic target and a prognostic biomarker [52].

Similarly, reductions in clostridial cluster XIVa species, including *Eubacterium rectale* and *Roseburia intestinalis*, have been strongly associated with increased CRC risk [49]. Sucralose-induced depletion of this cluster [28], may therefore reduce butyrate production and promote a pro-inflammatory, tumor-permissive environment.

A microbiome–CRC correlation analysis using ultra-deep shotgun metagenomic datasets from global microbiome surveys identified several Ruminococcus species as positively associated with CRC [53]. Consistent with this, Ruminococcus abundance is increased in the gut microbiota of CRC patients [54]. Chronic sucralose exposure in mice has also been reported to alter the intestinal abundance of this genus, suggesting that sucralose-induced dysbiosis may intersect with microbial signatures linked to CRC risk. However, current studies have not resolved these shifts at the species or subspecies level, limiting the ability to distinguish whether sucralose affects protective or pathogenic Ruminococcus taxa, a critical distinction given that subspecies-level resolution has proven essential for accurate CRC prediction.

Table 2 summarizes human intervention studies that evaluate the effects of sucralose intake on gut microbiota composition and related metabolic outcomes. Overall, the human literature remains limited by modest sample sizes, short exposure periods, heterogeneous formulations, and variable host metabolic backgrounds, all of which limit generalizability. Taken together, these findings suggest that sucralose-induced dysbiosis may overlap with microbial alterations reported in CRC and could influence metabolic and immunological pathways relevant to mucosal defense and antitumor immunity. However, these links remain largely inferential, as current evidence is derived predominantly from preclinical models and limited human datasets.

## 4. Barrier Dysfunction and Mucosal Inflammation

The integrity of the intestinal epithelial barrier relies on the proper organization of tight junctions—specialized multiprotein complexes that connect adjacent epithelial cells through transmembrane proteins. These structures form a dynamic seal that preserves both the physical and functional compartmentalization of the intestinal mucosa, preventing the uncontrolled passage of luminal contents into the lamina propria [58]. Maintenance of this barrier is therefore critical for mucosal homeostasis and for preventing inappropriate immune activation [59].

When barrier integrity is compromised, increased intestinal permeability facilitates the translocation of luminal contents into host tissues, promoting inflammation and contributing to the onset or exacerbation of inflammatory bowel diseases (IBDs) [60]. Recent attention has focused on sucralose-6-acetate, an intermediate and impurity generated during sucralose synthesis, which is present in commercial formulations at concentrations up to 0.67% [61]. In vitro studies using polarized human colonic epithelial cells grown on transwell systems demonstrated that both sucralose and sucralose-6-acetate disrupted epithelial permeability in human colonic monolayers at millimolar concentrations, even in the absence of bacterial components. Sucralose exposure (≤1000 µM, 24 h) in Caco-2 cells does not affect viability or apoptosis but reduces claudin-3 surface expression via a T1R3-dependent pathway, suggesting a potential for destabilizing tight junctions [62]. It is important to note that these findings represent in vitro observations, and discrepancies across studies may depend on experimental variables, such as exposure duration. Indeed, shorter incubation times with sucralose appear to have minimal or no effect on permeability changes or transepithelial electrical resistance (TEER) measurements in the same cell line [63].

In vivo, the impact of sucralose on gut barrier integrity is shaped by complex physiological variables, including luminal distribution, metabolic handling, and microbiota composition. Experimental studies in mice have shown that chronic exposure to sucralose, administered for up to 16 weeks at concentrations ranging from low doses to 0.3 mg/mL (comparable to the ADI established by the FDA), can induce significant alterations in intestinal integrity [40]. Chronic sucralose consumption induces epithelial barrier disruption, characterized by crypt damage, goblet cell loss, inflammatory infiltrates, and acute colitis, even at very low concentrations (0.0003 mg/mL) [28,40]. In OVA-sensitized mice, sucralose elicited duodenal inflammation with moderate villous blunting and marked architectural disorganization [64]. Rats exposed for 12 weeks to Splenda^®^, a commercial formulation containing sucralose and other additives, developed lymphocytic infiltration of the epithelium, epithelial scarring, goblet cell depletion, glandular disorganization, and focal vascular dilation with intravascular lymphocytes in the colon [65]. However, it is important to note that the presence of additional ingredients in Splenda^®^ complicates attributing these effects specifically to sucralose. By contrast, in healthy adults, acute intake of sucralose did not affect intestinal barrier function, as no bacterial endotoxin translocation was observed after consumption of 1 L of a sucralose-sweetened beverage [66], indicating that sucralose did not alter intestinal permeability in the short term.

Excessive intake of sucralose may be particularly harmful during pregnancy and lactation. Maternal sucralose exposure disrupts offspring intestinal development by inducing gut microbiota imbalance, promoting low-grade inflammation, and disrupting epithelial barrier function [42]. Expression of tight junction proteins (Claudin-1, Claudin-3, ZO-1) and secretory IgA is markedly reduced, while pro-inflammatory cytokines (IFN-γ, IL-1β, TNF-α) are increased [42]. These early-life alterations in microbial composition and mucosal immunity may establish a pro-inflammatory baseline that persists into adulthood. Supporting this, Aguayo-Guerrero et al. [67] observed a substantial increase in inflammatory non-classical monocytes, with increased IL-1β and TNF-α expression and diminished IL-10 production in 87 infants whose mothers had high sucralose intake.

In the gut, enteric neurons produce nitric oxide (NO) as an important neurotransmitter to evoke smooth muscle relaxation, allowing successful propulsion of luminal contents along the digestive system [68]. Although reactive oxygen species (ROS) and reactive nitrogen species (RNS) are required for normal physiological functions, disturbances in this delicate balance can lead to a highly pro-oxidative environment and oxidative stress. Oxidative stress in living organisms results from an imbalance between ROS production and the capacity to neutralize them. The disparity between excessive reactive molecules and weakened endogenous defenses leads to damage to cellular structures and biomolecules, such as lipids, proteins, and DNA, ultimately contributing to the pathogenesis of a wide range of diseases [69,70]. In the gastrointestinal system, this imbalance can alter epithelial function, intestinal motility, and microbiota homeostasis, favoring the development of various pathologies such as gastritis, colitis, and IBDs [71,72,73]. Gastrointestinal inflammation represents a complex immune response characterized by activation of the intestinal epithelium, infiltration of immune cells, and release of pro-inflammatory cytokines, including IL-6, TNF-α, and IL-1β. When sustained chronically, this process contributes to mucosal damage, loss of epithelial barrier integrity, and functional disturbances of the intestine [74]. The interaction between oxidative stress and inflammation is bidirectional: excessive ROS can activate pro-inflammatory pathways such as NF-κB, whereas persistent inflammation can enhance free radical production, perpetuating a self-reinforcing cycle of tissue injury.

Sucralose contributes to oxidative and inflammatory stress in vitro and in vivo. In an in vitro model, Hacioglu et al. [75] evaluated the effects of chronic sucralose exposure on human microglial cells (HMC3 line) to assess its impact on inflammation and oxidative stress. Cells were exposed to various concentrations of sucralose (0–50 mM) and to 1 mM for 21 days. Sustained sucralose exposure induced a time-dependent increase in inflammatory markers (IL-1β and NLRP3) and oxidative damage indicators (8-OHdG and MDA), exceeding 50% after 21 days of treatment. Morphological alterations consistent with structural damage—such as cytoplasmic shrinkage and cell rounding—were also observed, together with a significant reduction in migratory capacity in wound-healing assays. In rodent liver tissue, high-dose sucralose (3 g/kg/day) drives genomic instability and ROS accumulation [76], though the supraphysiological dosage limits direct translation to humans.

Multiple studies have demonstrated that sucralose consumption can exacerbate intestinal inflammation through both microbiota-dependent and independent mechanisms [27,62,77,78]. In murine models, long-term exposure to sucralose, even at concentrations comparable to the ADI, increases colonic infiltration of macrophages, neutrophils, and lymphocytes, accompanied by elevated expression of pro-inflammatory cytokines (TNF-α, IL-1β, and IL-6) [40,79,80,81]. These inflammatory mediators promote mucosal damage, impair epithelial regeneration, and amplify oxidative stress, thereby establishing a self-sustaining inflammatory loop.

Sucralose also alters innate and adaptive immune function. In macrophages, sucralose treatment induces intracellular Ca^2+^ depletion, triggering endoplasmic reticulum (ER) stress and impairing cell migration via taste receptor-dependent pathways [82]; however, bone marrow-derived macrophages cultured with sucralose did not exhibit altered production of IL-1β, IL-6, or IL-12p70 in response to LPS stimulation, suggesting that its effects on macrophages may be context- or activation-state-dependent [83].

In parallel, effector CD4^+^ and CD8^+^ T cells exposed to sucralose show reduced IFN-γ production and a transcriptional program characteristic of metabolic stress and early exhaustion [83]. Sucralose also affects antigen-presenting cells: in OVA-allergic mice, sucralose exposure markedly increased splenic MHC II and CD11c expression, indicating enhanced dendritic cell (DC) activation. Consistently, DCs treated with 1–5 mM sucralose for 24 h showed a robust, dose-dependent upregulation of CD80, CD86, and MHC II, and co-stimulation with OVA further amplified these maturation markers. These findings support that sucralose directly promotes DC maturation and synergizes with antigen exposure to potentiate antigen presentation and T-cell priming, potentially facilitating food sensitization [64].

Gómez-Arauz et al. [84] conducted a randomized, placebo-controlled, parallel clinical trial to assess the acute effects of sucralose on metabolic and immune parameters in healthy young adults. Participants received 48 mg of sucralose or a placebo 15 min before an oral glucose tolerance test. Although no significant differences in glucose levels were observed between groups, sucralose intake significantly increased serum insulin concentrations at 30, 45, and 180 min post-ingestion, suggesting a sweetener-induced hyperinsulinemic response. Moreover, alterations in monocyte subpopulations were detected, including a 7% increase in classical monocytes (CD14++CD16−) and a 63% reduction in non-classical monocytes (CD14+CD16+), together with decreased expression of CD11c and CD206, markers associated with anti-inflammatory and phagocytic functions. Collectively, these findings indicate that even acute exposure to sucralose can trigger an immunometabolic response characterized by hyperinsulinemia and pro-inflammatory shifts in mononuclear cells. Conversely, evidence from studies of microbiota-targeted interventions suggests that restoration of intestinal microbial homeostasis may improve glucose metabolism, reduce insulin resistance, and strengthen barrier integrity. In this context, nutritional compounds containing saponins, flavonoids, and polysaccharides have been reported to exert beneficial effects on intestinal permeability and glycemic control, further supporting the link between microbiota composition, metabolic regulation, and inflammatory tone [85]. Together, the convergence of epithelial stress, innate immune activation, and adaptive T-cell dysfunction may create a microenvironment of chronic inflammation, immune dysregulation, and increased tumor-promoting potential.

Experimental evidence shows that exposure to sucralose differentially affects colitis severity depending on dose and duration. Low-dose sucralose (0.1 mg/mL for 21 days) did not exacerbate dextran sodium sulfate (DSS)-induced colitis in mice and was even associated with a modest reduction in the disease activity index (DAI) [38]. In contrast, higher doses have consistently produced detrimental effects. Mice exposed to 1.5 mg/mL sucralose for 6 weeks exhibited pronounced weight loss, elevated DAI scores, and increased plasma cell infiltration and lymphocyte aggregation in the DSS colitis model. Mechanistically, the sucralose + DSS group showed significant upregulation of TLR5 and NF-κB, along with enhanced production of pro-inflammatory mediators—including TNF-α, IL-1β, IL-18, IL-17A, IL-22, and the activation marker NLRP3—and expansion of Th2 and Th17 populations. Conversely, anti-inflammatory pathways were suppressed, as evidenced by reduced IL-10, NLRP12, and Th1 cell frequency [81].

**Figure 1:**
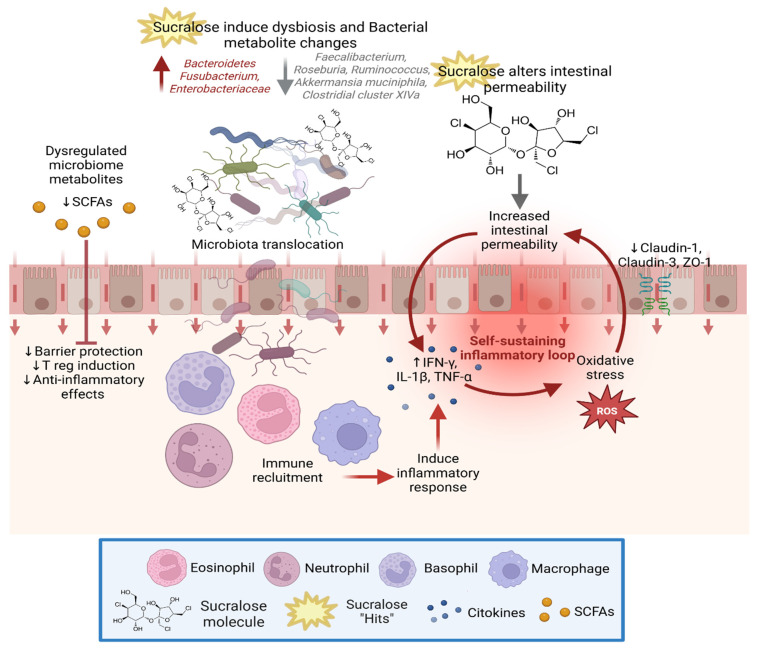
Proposed mechanisms by which sucralose exposure may disrupt intestinal homeostasis, based primarily on preclinical studies. Sucralose exposure may impair epithelial barrier integrity and alter tight junction protein expression, potentially increasing intestinal permeability and microbial translocation into the lamina propria. This process may trigger immune activation and promote a pro-inflammatory environment. In parallel, sucralose-induced dysbiosis may favor the expansion of taxa such as Bacteroidetes, Fusobacterium, and Enterobacteriaceae, further amplifying inflammation through increased production of cytokines (e.g., IFN-γ, TNF-α, IL-1β) and oxidative stress. Additionally, altered microbial metabolism may reduce levels of protective metabolites, and short-chain fatty acids (SCFAs), which normally support barrier integrity and immune regulation. Together, these alterations compromise intestinal homeostasis and may increase susceptibility to inflammatory bowel disease (IBD) and colorectal cancer (CRC). ↑ indicates increased abundance or levels. ↓ indicates decreased abundance or levels. Created in BioRender.com Mejia Muñoz, A. (2026) https://app.biorender.com/illustrations/69a0cf44005c80734df2203c?slideId=99b4e5ac-d296-430b-ae3d-4bf778ce9fca (accessed on 2 February 2026).

The azoxymethane/DSS (AOM/DSS) model of colitis-associated colorectal cancer showed a similar pro-inflammatory profile. Sucralose exposure increased both the number and size of colorectal tumors and affected multiple pathological parameters, including body and spleen weight, histological injury scores, fecal β-glucuronidase and protease activity, epithelial barrier markers, gut microbiota composition, and cytokine expression. These changes were linked to activation of the TLR4/MyD88/NF-κB and STAT3/VEGF signaling pathways, which are known to promote tumors in chronic colitis [34]. Overall, these results suggest that sucralose exposure may affect epithelial integrity, signaling pathways, and immune responses in preclinical models, potentially contributing to a mucosal environment associated with chronic inflammation and an increased risk of colitis-associated tumor development. However, these effects appear to depend on dose, formulation, duration, and experimental conditions (Figure 1).

## 5. Potential Effects of Sucralose on the Tumor Microenvironment and T-Cell Function

The findings reported by Zani et al. [83] indicate that exposure to sucralose in mice attenuates the differentiation of both CD4^+^ and CD8^+^ T cells toward IFN-γ-producing effector lineages, suggesting a dampened type-1 immune response. Mechanistically, sucralose interferes with T-cell receptor (TCR) signaling, impairing TCR-dependent proliferation by disrupting PLCγ1 microcluster formation and reducing endoplasmic reticulum-mediated calcium release. Moreover, alterations in membrane order and fluidity induced by sucralose may further hinder the assembly of signaling complexes required for effective T-cell activation. To assess the impact of sucralose on T-cell-mediated immunity in vivo, Zani et al. [83] investigated tumor-specific immune responses in a murine cancer model. Mice receiving high concentrations of sucralose in drinking water (0.72 mg/mL) exhibited accelerated growth of subcutaneous EL4-OVA tumors compared with controls. This effect was associated with a marked reduction in the antitumor activity of CD8^+^ effector T cells, primarily due to decreased IFN-γ production. Adoptive transfer experiments using OVA-specific OT-I T cells confirmed that sucralose intake suppressed tumor-specific cytotoxic responses, thereby impairing immune-mediated tumor control. Consistent with these in vivo findings, in vitro activation of OT-I cells in the presence of sucralose reduced cytotoxic activity against EL4-OVA targets. Collectively, these findings indicate that sucralose can modulate T-cell function at multiple levels, potentially weakening adaptive immune responses and influencing the balance between pro-inflammatory and regulatory mechanisms within the intestinal mucosa.

More broadly, growing evidence indicates that the gut microbiome can shape the tumor microenvironment and may offer a therapeutic target for enhancing antitumor immunity. In this context, microbiome-based strategies have been demonstrated to affect immune cell infiltration, inflammatory signaling, and tumor-related immune responses, supporting the idea that microbial composition is not only associated with tumor progression but also a potential factor in modifying therapeutic responses [86].

To investigate whether sucralose may directly weaken effector T-cell responses necessary for optimal antitumor immunity and the activity of immune checkpoint inhibitors (ICIs), Morder et al. [87] recently reported an association between sucralose intake and impaired immunotherapy responses. They observed that sucralose intake was associated with impaired responses to immunotherapy in preclinical models and worse outcomes in non-CRC patient groups. The investigators enrolled patients with advanced metastatic cutaneous melanoma or non-small-cell lung cancer (NSCLC) who were scheduled to receive systemic anti-PD-1-based immunotherapy or chemoimmunotherapy in a prospective registry and assessed their sucralose intake. The study showed a significant negative association between high sucralose intake (>0.16 mg/kg/day) and clinical outcomes in patients receiving ICI. In both melanoma and NSCLC groups, elevated sucralose consumption was associated with lower overall response rates (ORRs) and shorter progression-free survival (PFS). To explore the potential mechanism underlying resistance to ICI therapy, they treated Taconic mice with 0.09 mg/mL sucralose for 14 days. They found that mice consuming sucralose were resistant to PD1 blockade in both MC38 (adenocarcinoma) and B16 (melanoma) models, exhibiting significantly increased tumor growth, decreased CD8+ T-cell infiltration, and reduced survival. Similarly, in a CAC model, sucralose-consuming mice showed significantly increased tumor burden and size. Given these findings, the authors conducted RNA sequencing to investigate potential mechanisms. SLIDE (Significant Latent Factor Interaction Discovery and Exploration) revealed that CD8 T cells from mice exposed to sucralose during anti-PD-1 treatment exhibited gene signatures associated with severe dysregulation or potential exhaustion. RNA-seq enrichment analysis identified several affected pathways, including those related to metabolic stress and exhaustion. The authors subsequently conducted additional experiments to determine the direct effects of sucralose on T-cell function and to evaluate whether the timing of exposure influenced T-cell activity. CD8+ T cells expanded in media supplemented with sucralose proliferated more slowly, exhibited increased apoptosis, showed reduced Granzyme B expression, and had a diminished ability to kill target cells in vitro compared to untreated controls [87]. Collectively, these results suggest that excessive consumption of sucralose can impair CD8^+^ T-cell effector functions and weaken antitumor immunity. Therefore, available data imply that sucralose might affect T-cell function in experimental systems, although its effects in patients remain unconfirmed.

## 6. Could Sucralose Influence Immunotherapy Efficacy? Translational Considerations for Colorectal Cancer

ICIs have shown efficacy in clinical trials, with nivolumab, pembrolizumab, and ipilimumab approved by the FDA for CRC patients with microsatellite instability-high (MSI-H) or mismatch repair-deficient (dMMR) [88,89]. Recent biomarker analyses from the CheckMate 142 trial have improved our understanding of how inflammation-related gene expression signatures (GESs) and tumor mutational burden (TMB) differ in their predictive value for the efficacy of ICI in MSI-H/dMMR colorectal cancer. In this study, high inflammatory levels and tertiary lymphoid structures (TLSs) (GES scores) were linked to better progression-free and overall survival among patients treated with nivolumab alone [88], suggesting that a primed immune microenvironment capable of supporting T-cell infiltration and TLS formation is necessary for effective immunotherapy. However, dietary and environmental factors that influence intestinal immunity and microbial metabolism, such as sucralose intake, might alter the key determinants of the response.

Perturbations in the gut microbiome can significantly influence both cancer development and responses to immunotherapy [90,91]. Increasing evidence indicates a strong association between high gut microbial diversity and improved clinical outcomes following ICI [91,92,93,94,95]. In a recent study, fecal microbiota transplantation (FMT) from responder (R) and non-responder (NR) cancer patients into mice with Lewis lung carcinoma tumors demonstrated that FMT from responders notably enhanced the efficacy of PD-1 blockade and decreased the Ki-67 tumor proliferation marker [91]. In mice, high blood levels of butyrate and propionate are associated with resistance to CTLA-4 blockade and a greater proportion of Treg cells. Elevated blood butyrate levels in patients seem to suppress ipilimumab-induced growth of memory and ICOS^+^ CD4^+^ T cells, as well as IL-2–related immune activation [96]. These findings suggest that the diversity and functional capacity of the gut microbiota—especially its ability to produce SCFAs—are crucial for successful immunotherapy. On the other hand, microbial imbalance can promote chronic inflammation and support an environment that encourages tumor growth.

After sucralose consumption, Firmicutes and Proteobacteria phyla increased, with a relative outgrowth of select Gram-positive bacteria, including Clostridiaceae and Lachnospiraceae [87]. During anti-PD-1 treatment, levels of arginine, as well as many associated metabolites (citrulline), were significantly reduced in the stool of sucralose-consuming mice. Arginine is a key metabolite required for optimal T-cell metabolism and function, and it has been previously associated with cytotoxic T-cell function in cancer. Interestingly, citrulline supplementation restored the response to anti-PD-1 even in the presence of sucralose. In MSS CRC-bearing mice, antibiotic-induced disruption of the gut microbiome significantly reduced the antitumor efficacy of PD-1 blockade, demonstrating that microbiome composition and associated metabolic pathways—particularly glycerophospholipid metabolism, which regulates IFN-γ and IL-2 production—are key determinants of therapeutic response. These findings suggest that diet-induced dysbiosis, including alterations associated with chronic sucralose consumption, may influence biological pathways relevant to the immunotherapy response. However, whether sucralose exposure represents a clinically meaningful modifier of treatment efficacy in CRC patients remains unknown [97]. Importantly, the currently available human data linking sucralose intake with immunotherapy outcomes come from melanoma and NSCLC cohorts, not from patients with CRC.

In tumor-bearing mice, sucralose intake diminished anti-PD-1 responses, resulting in larger tumors, fewer CD8^+^ T cells, and poorer survival. Mechanistically, sucralose exposure in preclinical models was associated with alterations in the gut microbiome and impaired T-cell metabolism and function, including exhaustion-related programs and reduced IFN-γ production, which were linked to decreased responsiveness to checkpoint blockade [87]. Importantly, this resistance depended on the microbiome. Fecal transfer from sucralose-exposed donors replicated the resistance; transferring feces from responder mice restored the efficacy of anti-PD-1. Supplementing with citrulline/arginine rescued T-cell function and responses, indicating that microbial arginine breakdown is a key factor [87].

Recent controlled human studies have shown that sucralose is not metabolically inert. In a randomized trial involving healthy adults naive to non-nutritive sweeteners, two weeks of sucralose consumption (at doses below the acceptable daily intake) significantly impaired glucose tolerance compared with controls [43]. Multi-omics analyses revealed that sucralose changed gut and oral microbiome composition and metabolic pathways, especially purine metabolism. These microbiome changes were functionally connected to host physiology, as fecal transplants from “sucralose responders” to germ-free mice recreated the glucose intolerance phenotype, confirming a microbiome-mediated causal mechanism. Notably, the metabolic effects of sucralose varied greatly among individuals, depending on their baseline microbial composition and function.

In contrast, Ahmad et al. and Thomson et al. [55,57] reported that short-term, low-dose sucralose exposure had minimal effects on the microbiome and metabolic markers in healthy adults. However, these studies used 16S rRNA sequencing, which limits taxonomic resolution, whereas Suez et al. [43] employed shotgun metagenomics and metatranscriptomics, revealing deeper functional disruptions at the metabolic pathway level. Collectively, these findings suggest that habitual sucralose intake, even at regulatory “safe” levels, may disrupt host–microbe interactions and predispose susceptible individuals to metabolic dysregulation.

**Figure 2:**
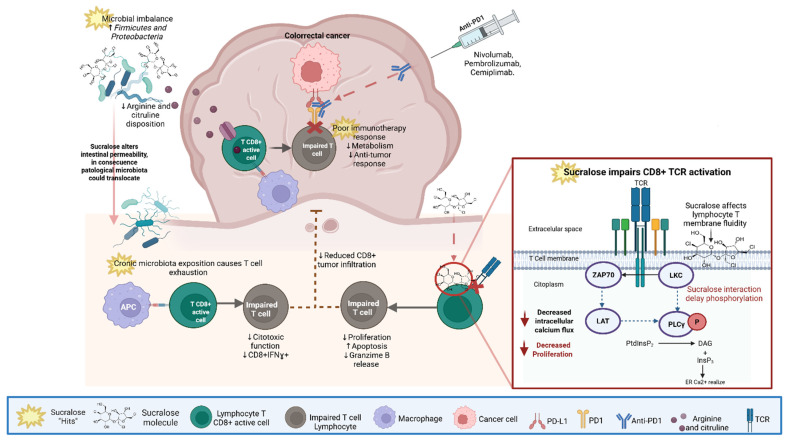
Proposed mechanisms by which sucralose-associated microbiome alterations may influence antitumor immunity and responsiveness to immune checkpoint inhibitors, based primarily on preclinical evidence. Dysbiosis may reduce the availability of key microbial metabolites, including arginine and citrulline, which are required for optimal CD8^+^ T-cell metabolism, immune infiltration, and cytotoxic activity. Within the tumor microenvironment, this metabolic limitation contributes to impaired T-cell fitness and reduced responsiveness to immune checkpoint inhibitors. In parallel, chronic immune exposure to dysbiotic microbiota promotes inflammatory signaling and T-cell exhaustion. Sucralose may also directly interfere with T-cell receptor (TCR) signaling by altering membrane organization and delaying phospholipase C-γ (PLCγ) phosphorylation, thereby impairing T-cell activation and proliferation. ↑ indicates increased abundance or levels. ↓ indicates decreased abundance or levels. Dotted lines indicate inhibition. Created by BioRender.com Mejia Muñoz, A. (2026) https://app.biorender.com/illustrations/6737e57da1b13aabf52cf183?slideId=99b4e5ac-d296-430b-ae3d-4bf778ce9fca (accessed on 2 February 2026).

Collectively, these findings suggest that sucralose consumption may represent a previously underrecognized, yet potentially modifiable, factor influencing immunotherapy efficacy. By altering the gut microbiome and associated metabolic pathways in preclinical models, sucralose exposure may influence immune features relevant to checkpoint blockade responsiveness. Whether these observations translate into clinically meaningful effects in CRC patients remains unknown (Figure 2).

Although most mechanistic evidence currently derives from preclinical models, emerging human data indicating microbiome and metabolic alterations even at intake levels below the acceptable daily intake underscore the need for caution. Importantly, the microbiome-dependent reversibility of these effects—demonstrated through fecal transfer and metabolic supplementation strategies—suggests that dietary modulation and microbial-targeted interventions could enhance immunotherapy responsiveness.

These observations highlight the importance of considering dietary exposures such as non-nutritive sweeteners as integral components of the tumor–immune ecosystem. Prospective clinical studies are now required to determine whether sucralose intake influences therapeutic outcomes in patients receiving ICI, including those with MSI-H/dMMR CRC, and whether nutritional or microbiome-based interventions could mitigate these effects. Accordingly, the relevance of these findings to CRC should be regarded as translationally plausible but not clinically demonstrated.

## 7. Clinical and Translational Implications of Chronic Sucralose Consumption

Sucralose has been appraised primarily through traditional toxicological paradigms, from which ADI thresholds were derived. These frameworks remain indispensable for safety assessment but are not designed to interrogate inter-individual variability driven by host–microbiome interactions. The translational question is whether sustained sucralose exposure can reproducibly alter microbial function, thereby reshaping mucosal immune tone, inflammatory signaling, and, in defined settings, treatment responsiveness.

Controlled human data move this discussion beyond plausibility. In a randomized trial involving healthy adults, short-term exposure to non-nutritive sweeteners, including sucralose, at doses below ADI produced individualized metabolic responses that tracked with microbiome remodeling, supporting a causal contribution of microbial changes to host phenotypes [43]. Two implications follow. First, biological inertness should not be assumed at typical exposure ranges. Second, heterogeneity is intrinsic to the response and should be treated as an effect modifier to be explained, not noise to be averaged out.

For colorectal disease, preclinical data support prioritizing inflammation- and barrier-vulnerable contexts in prospective studies. In mice, sucralose exacerbated DSS-induced colitis and increased colitis-associated colorectal cancer burden, accompanied by dysbiosis and barrier-related perturbations [34]. While these findings do not directly translate into population-level cancer risk estimates, they provide a mechanistic rationale for examining sucralose exposure in strata with baseline dysbiosis and chronic immune activation, including inflammatory bowel disease, colitis phenotypes, obesity with metabolic dysfunction, and established colorectal neoplasia.

The most immediate high-stakes implications lie in immuno-oncology. Seminal clinical studies show that gut microbiome configuration influences outcomes with programmed cell death protein 1 (PD-1)-based immunotherapy, and that microbiome disruption—particularly antibiotic exposure—is frequently associated with poorer responses [98]. Against this background, recent translational work identifies sucralose as a candidate modifiable exposure that may compromise checkpoint blockade via microbiome-dependent mechanisms. Morder et al. [87] recently reported an association between sucralose intake and impaired immunotherapy responses in preclinical models and non-CRC patient cohorts. Higher sucralose consumption was associated with worse outcomes in patients receiving anti-PD-1 regimens; in tumor-bearing mice, sucralose exposure attenuated anti-PD-1 efficacy, paralleled by impaired CD8^+^ T-cell fitness. Mechanistic analyses implicated microbiome destabilization with reduced microbiota-accessible arginine and an immune–metabolic state consistent with cytotoxic T-cell dysfunction. Notably, citrulline/arginine supplementation and fecal microbiome transfer restored T-cell function and therapeutic response [87]. The value of this work lies in its tight integration of exposure, mechanism, and an actionable metabolic node, providing a concrete template for prospective validation.

This evidence supports treating sucralose exposure as a quantified variable in immunotherapy trials and translational cohorts rather than a binary “user/non-user” attribute. Exposure ascertainment should capture the estimated dose, duration, and timing relative to therapy initiation, with prespecified adjustments for major microbiome modifiers such as antibiotics, proton pump inhibitors, metformin and related metabolic agents, baseline dietary patterns (including fiber), obesity/metabolic status, and inflammatory comorbidities. Endpoints should extend beyond taxonomy to functional microbial readouts aligned with the proposed mechanisms, including pathways related to short-chain fatty acid production and amino acid metabolism (arginine/citrulline), paired with immune phenotyping focused on CD8^+^ activation state, exhaustion-associated programs, and effector function. Metabolite-level endpoints are especially attractive given the precedent for microbiome-derived metabolites shaping checkpoint inhibitor responses and offering intervention leverage points [99].

Interventional translation should remain evidence-led. Current data do not support universal clinical restriction of sucralose, nor do they justify prescriptive recommendations in routine practice in the absence of prospective, clinically anchored trials. They do, however, justify targeted study designs in settings where outcomes depend on cytotoxic T-cell competence and where microbiome-mediated variability is well established. Practical approaches include controlled dietary run-in periods before and during anti-PD-1 therapy with prespecified functional microbial and immune endpoints, as well as rigorously phenotyped pragmatic cohorts embedded in routine care.

Overall, the clinical implication is a reframing of sucralose from an unmeasured dietary detail to a quantifiable exposure with plausible microbiome–immune consequences in defined contexts. A precision nutrition framework integrating dietary exposures, microbial function, and immune competence provides a disciplined path to determine whether, in whom, and under what exposure patterns sucralose-related microbiome remodeling becomes clinically meaningful.

## 8. Conclusions

Taken together, the available evidence suggests that sucralose may aggravate intestinal inflammation and favor colitis-associated tumorigenesis in susceptible settings. In murine DSS and AOM/DSS models, sucralose exposure has been linked to gut dysbiosis, depletion of beneficial SCFA-producing bacteria, disruption of epithelial barrier-related molecules, increased inflammatory cytokine production, and activation of tumor-promoting pathways, including TLR4/MyD88/NF-κB and STAT3/VEGF. These alterations are associated with more severe colitis, increased tumor burden, and greater histopathological damage in several preclinical studies, although outcomes vary according to dose, formulation, duration of exposure, and experimental context.

Emerging evidence also indicates that sucralose may influence antitumor immunity. In preclinical models, sucralose impairs CD8^+^ T-cell fitness, reduces IFN-γ production, and promotes immune–metabolic programs associated with T-cell dysfunction, changes that coincide with weaker antitumor responses and reduced efficacy of immune checkpoint blockade, mediated by microbiome-dependent mechanisms. Thus, beyond its effects on mucosal inflammation and barrier integrity, sucralose may also modulate tumor–immune interactions relevant to immunotherapy responsiveness.

However, the current evidence base remains limited. Most mechanistic data derive from animal models and in vitro systems, whereas human studies are few, frequently small, and heterogeneous in exposure assessment, formulation, duration, host characteristics, and endpoints. In addition, findings from pure sucralose, commercial mixtures, and sucralose-related compounds such as sucralose-6-acetate should not be considered interchangeable. Human evidence linking sucralose intake with impaired immunotherapy outcomes currently derives from non-CRC cohorts; therefore, its relevance to colorectal cancer should be regarded as translationally plausible rather than clinically demonstrated. Overall, the current literature should be interpreted as hypothesis-generating and translationally informative rather than clinically definitive, and prospective human studies are needed to determine whether these preclinical observations translate into clinically meaningful risk.

## Figures and Tables

**Table 1:** Summary of preclinical studies evaluating the effects of sucralose on gut microbiota composition, microbial metabolites, and related biological outcomes.

Methodology *	Sucralose Dose	Estimated HED **	Microbiota Analysis Tool	Gut Microbiota Alterations	Microbiota-Related Metabolite Alterations	Other Results	Reference
C57BL/6J male mice (~8 weeks old; *n* = 10/group) receive sucralose in drinking water for 6 months.	0.1 mg/mL	~1.30 mg/kg/day	16S rRNA gene sequencing (V4) Functional gene enrichment analysis Fecal metabolomic analysis	Sucralose group vs. control group: ↑ Lachnospiraceae *Ruminococcus*, Streptococcaceae *Streptococcus*, Lachnospiraceae *Anaerostipes*, Staphylococcaceae *Staphylococcus*, Peptostreptococcaceae, Erysipelotrichaceae, and Bacillales ↓ Ruminococcaceae *Ruminococcus*, Turicibacteraceae *Turicibacter*, Lachnospiraceae *Roseburia*, Verrucomicrobiaceae *Akkermansia*, Clostridiaceae, and Christensenellaceae	↓ Abundance of quorum-sensing signals Disturbed amino acid fecal metabolites involved in tryptophan metabolism ↓ bile acids with antimicrobial effects	Sucralose treatment increases the expression of pro-inflammatory mediators, bacterial genes, and bacterial toxin genes, and elevates hepatic mRNA expression of inflammatory markers.	[27]
C57BL/6J mice (~6 weeks old; *n* = 5–9/group) receive sucralose in drinking water for 6 weeks during AOM/DSS induction.	1.5 mg/mL	~19.50 mg/kg/day	qRT-PCR for fecal bacteria analysis Determination of fecal β-glucuronidase, trypsin, and chymotrypsin activity	Sucralose/AOM/DSS group vs. AOM/DSS group: ↑ Firmicutes, Actinomycetes, *Peptostreptococcus stomatis*, *Clostridium symbiosum*, and *Peptostreptococcus anaerobius* ↓ Proteobacteria	Altered digestive proteases, with: ↑ fecal trypsin and chymotrypsin levels and ↓ fecal β-glucuronidase levels	Sucralose increased both tumor number and tumor size in the AOM/DSS model, with an 87.5% tumor incidence in the AOM/DSS + sucralose group versus 50% in the AOM/DSS group. Sucralose also increased TNF-α, TLR4, and MyD88 expression, while reducing IL-10, IκBα, and TRAF6 levels.	[34]
C57BL/6J female mice (~6 weeks old; *n* = 10/group) receive sucralose in drinking water for 11 weeks, with additional treatment arms including metformin or fructo-oligosaccharides. Other NNS are evaluated in separate experimental groups and are not administered simultaneously. *	0.1 mg/mL	~1.30 mg/kg/day	16S rRNA gene sequencing (V3–V4) Shotgun metagenomic sequencing Quantification of tryptophan metabolites and long-chain fatty acids (LCFAs)	Sucralose group vs. control group: ↑ Proteobacteria, Actinobacteria, *Streptococcus*, *Prevotella*, *Fusibacter*, *Lachnospira*, *Anaerovorax*, *Psychrilyobacter*, *Psychromonas*, and *Trabulsiella* ↓ Verrucomicrobia, *Lactobacillus*, and *Akkermansia muciniphila*	↑ Saturated fatty acids ↓ AhR agonists and microbiota-derived AhR ligands ↑ Fecal kynurenine	The sucralose group shows increased gut permeability, serum LPS, IL-6, and hepatic pro-inflammatory cytokines. In addition, mice develop lipid accumulation.	[35]
Diabetogenic C57BL/6 male mice (~7 weeks old; *n* = 8/group) receive sucralose in drinking water for 4 weeks. Other NNS are evaluated in separate experimental groups, not administered simultaneously.	48 mg/kg/day	~3.88 mg/kg/day	16S rRNA gene sequencing (V3–V4) Fecal metabolite analysis	Sucralose diabetogenic group vs. diabetogenic group: ↑ Firmicutes, Actinobacteriota, *Dubosiella*, and *Desulfovibrio* ↓ Bacteroidota and unclassified member of the family Muribaculaceae	Dysregulation of glycerophospholipid and choline metabolites	The diabetogenic sucralose-treated group shows reduced NF-κB mRNA levels.	[36]
Wistar male rats (~6 weeks old; *n* = 6–7/group) receiving an HFD are exposed to sucralose in drinking water for 4 months. Other NNS are evaluated in separate experimental groups, not administered simultaneously.	1.5% solution	~243 mg/kg/day	16S rRNA gene sequencing (V3–V4) Shotgun metagenomic sequencing	Sucralose/HFD group vs. sucralose group: ↑ Bacteroidetes, *Desulfovibrio*, *Enterococcus*, and *Butyricimonas* ↓ Firmicutes and *Akkermansia*	Enrichment of bacterial genes involved in LPS and SCFA synthesis	In HFD-fed animals, sucralose increases SCFAs, GPR43, TLR4, TNF-α, NF-κB, and LPS levels. In contrast, sucralose reduces sIgA and IL-10 levels.	[37]
C57BL/6J male mice (6–8 weeks old; *n* = 8/group) receive sucralose in drinking water for 4 weeks; DSS (2.5%) is administered from day 21 to induce colitis.	0.1 mg/mL	~1.30 mg/kg/day	16S rRNA gene sequencing (V3–V4)	Sucralose/DSS group vs. DSS group: ↑ Firmicutes, Bacteroidetes, *Ruminiclostridium*, *Mucispirillum*, *Bacteroides*, *Alistipes*, and *Akkermansia*	Not evaluated	Sucralose administration ameliorates DSS-induced colitis while promoting epithelial barrier recovery.	[38]
SAMP, AKR, and C57BL/6J mice (*n* = 6/group) receive incremental doses of Splenda^®^ in drinking water for 6 weeks. Doses are tested in three separate experimental arms in SAMP mice, which spontaneously develop ileitis.	Experiment 1: 1.08 mg/mL Experiment 2: 3.5 mg/mL Experiment 3: 35 mg/mL	Experiment 1: ~14 mg/kg/day Experiment 2: ~45 mg/kg/day Experiment 3: ~453 mg/kg/day	Metagenomic shotgun DNA sequencing 16S rRNA gene sequencing	SAMP/Splenda^®^ group vs. control group: ↑ Proteobacteria and *Escherichia coli* ↓ Chloroflexi, TM7, and *Streptococcus*	Not evaluated	In SAMP mice, Splenda^®^ treatment promotes replacement of *Streptococcus* spp. by *E. coli*, increases MPO activity, and enhances bacterial invasion into villous tissue.	[29]
C57BL/6J mice (~8 weeks old; *n* = 10/group) receive sucralose in drinking water for 6 months.	0.1 mg/mL	~1.30 mg/kg/day	16S rRNA gene sequencing	Sucralose group vs. control group: ↓ *Lactobacillus* and *Ruminococcus*	↓ Richness of bile salt hydrolase genes and of the secondary bile acid synthesis pathway Altered ratios of free bile acids and taurine-conjugated bile acids ↓ LCA, DCA, and CDCA levels in liver samples	Sucralose decreases FXR agonists, upregulates genes associated with lipogenesis, cholesterol absorption, and cholesterol efflux in the liver, and increases hepatic cholesterol levels.	[39]
C57BL/6J male mice (~4 weeks old; *n* = 8/group) receive sucralose for 16 weeks. Each dose is evaluated in a separate experiment.	Experiment1: 0.0003 mg/mL Experiment 2: 0.003 mg/mL Experiment3: 0.03 mg/mL Experiment 4: 0.3 mg/mL	Experiment 1: 0.0038 mg/kg/day Experiment 2: 0.038 mg/kg/day Experiment 3: 0.38 mg/kg/day Experiment 4: 3.88 mg/kg/day	16S rRNA gene sequencing (V4–V5)	Sucralose-treated groups vs. control group: ↑ Firmicutes, *Tenacibaculum*, *Ruegeria*, *Staphylococcus*, and *Corynebacterium* ↓ *Lachnoclostridium* and Lachnospiraceae	Not evaluated	T1 and T4 show severe acute colitis, with crypt destruction, loss of superficial epithelial cells and goblet cells, and increased inflammatory cell infiltration. Intermediate doses (T2 and T3) produce less severe effects than T1 and T4.	[40]
C57BL/6J male mice (~4 weeks old; *n* = 8/group) receive either a low or high dose of sucralose for 8 weeks. Other NNS are evaluated in separate experimental groups and are not administered simultaneously.	Low dose: 1.5 mg/kg/day High dose: 15 mg/kg/day	Low dose: 0.12 mg/kg/day High dose: 1.21 mg/kg/day	16S rRNA gene sequencing (V2–V3) Bile acid composition analysis Cecal metabolome analysis	Sucralose-treated groups vs. control group: ↓ Clostridium cluster XIVa	↑ Primary bile acids, especially the ratio of CA/CDCA, and the ratio of secondary bile acids (DCA and LCA) to primary bile acids (CA and CDCA) ↓ Butyrate concentration	Sucralose treatment increased hepatic cholesterol concentration.	[28]
C57BL/6 female mice (~8 weeks old; *n* = 17/group) receive sucralose during gestation and lactation, and offspring are evaluated after exposure.	0.1 mg/mL	~1.30 mg/kg/day	16S rRNA gene sequencing	3-week-old sucralose-exposed offspring vs. control group: ↑ Verrucomicrobia, Proteobacteria, *Akkermansia*, *Blautia*, *Corynebacterium*, and *Robinsoniella* ↓ Bacteroidetes, *Alistipes*, *Barnesiella*, *Paraprevotella*, Saccharibacteria (incertae sedis), and *Streptococcus*	↓ Butyrate production	Sucralose-exposed offspring show increased IL-1β, IFN-γ, and TNF-α levels, together with reduced GPR43 expression in colon tissue. HFD supplementation in exposed offspring promotes hepatic steatosis.	[41]
C57BL/6 female mice (~8 weeks old; *n* = 5–7/group) receive sucralose during gestation and lactation, and offspring are evaluated after exposure.	0.1 mg/mL	~1.30 mg/kg/day	16S rRNA gene sequencing	3-week-old sucralose-exposed offspring vs. control group: ↑ *Parabacteroides*, *Akkermansia*, *Blautia*, Desulfovibrionales, *Helicobacter*, Pasteurellales, and Campylobacterales ↓ *Bacteroides*, Clostridium XIVa, and *Parasutterella* 8-week-old sucralose-exposed offspring vs. control group: ↑ Firmicutes, *Alloprevotella*, *Parasutterella*, *Anaeroplasma*, and *Flavonifractor* ↓ Bacteroidetes, *Alistipes*, *Barnesiella*, and *Butyricicoccus*	↓ Expression of antimicrobial peptides such as cryptdins and lysozymes	Sucralose exposure inhibits intestinal development, increases pro-inflammatory cytokine expression in the small intestine, increases abnormal Paneth cell granule secretion, and reduces Paneth cell number.	[42]

Abbreviations: AhR, aryl hydrocarbon receptor; AOM, azoxymethane; CA, cholic acid; CDCA, chenodeoxycholic acid; DCA, deoxycholic acid; DSS, dextran sodium sulfate; FXR, farnesoid X receptor; HED, human-equivalent dose; HFD, high-fat diet; IL, interleukin; LCA, lithocholic acid; LCFAs, long-chain fatty acids; LPS, lipopolysaccharide; MPO, myeloperoxidase; NF-κB, nuclear factor kappa B; NNS, non-nutritive sweeteners; SCFAs, short-chain fatty acids; sIgA, secretory immunoglobulin A; TLR, Toll-like receptor; TNF-α, tumor necrosis factor alpha. * The reported results correspond to sucralose exposure and do not account for the independent effects of alternative NNS when these were evaluated in the same study. Results obtained with commercial sucralose-containing formulations (e.g., Splenda^®^) should not be interpreted as equivalent to those obtained with pure sucralose, as these products may contain additional compounds that influence biological outcomes. ** Estimated HED values are provided to facilitate comparison with the FDA acceptable daily intake (ADI) for sucralose (5 mg/kg/day). These estimates should be interpreted cautiously, as cross-study comparisons are influenced by differences in species, formulation, route of administration, and exposure duration. ↑ indicates increased abundance or levels relative to the corresponding comparison group. ↓ indicates decreased abundance or levels relative to the corresponding comparison group.

**Table 2:** Summary of human intervention studies evaluating the effects of sucralose on gut microbiota composition and related metabolic outcomes *.

Study Type	Subjects	Methodology	Sucralose EDI **	Microbiota Analysis	Gut Microbiota Alterations	Other Results	Reference
Randomized, double-blind, crossover controlled clinical trial	Healthy adults aged 18–45 years (*n* = 17)	Participants consume 136 mg/day of sucralose or 425 mg/day of aspartame for 12 weeks. During weeks 5–6, half of the participants consume aspartame (*n* = 9) and the remainder consume sucralose (*n* = 8). During weeks 7–10, participants consume no NNS. During weeks 11–12, all participants consume the sweetener not previously assigned.	~2.0 mg/kg/day Below FDA ADI (0.4×)	16S rRNA gene sequencing (V4)	Sucralose arm, pre-treatment vs. post-treatment: No significant differences in the median relative abundance of the most prevalent bacterial taxa were reported.	Sucralose and aspartame do not alter fecal metabolites or overall microbiota composition.	[55]
Open-label randomized clinical trial	Healthy adults aged 18–35 years (*n* = 40)	Participants consume 48 mg/day of sucralose (*n* = 20) or pure water (*n* = 20) for 10 weeks.	~0.8 mg/kg/day Below FDA ADI (0.16×)	RT-qPCR for selected bacteria	Sucralose arm vs. control group: ↑ *Blautia coccoides* ↓ *Lactobacillus acidophilus*	Participants in the sucralose group show increased insulin levels.	[56]
Two open-label, parallel-arm randomized controlled trials	Trial 1: Adults with T2DM aged 30–50 years (*n* = 49) Trial 2: Adults with overweight or obesity aged 25–50 years (*n* = 48 ***)	Participants in the intervention arm of both trials consume 6 mg/day of sucralose for 12 weeks. Control participants maintain their usual diet without sucralose supplementation.	~0.1 mg/kg/day Below FDA ADI (0.02×)	16S rRNA gene sequencing (V3–V4)	Trial 1, sucralose arm vs. control group: ↑ *Enterococcus* and *Pediococcus* ↓ Firmicutes, *Agathobacter*, *Lachnoclostridium*, *Lachnospira*, *Megamonas*, *Faecalibacterium*, *Roseburia*, and *Fusicatenibacter* Trial 2, sucralose arm vs. control group: ↓ Firmicutes, *Enterococcus*, and Clostridia	Adults with T2DM show decreased α-diversity and increased β-diversity in gut microbiome communities.	[31]
Pilot dietary intervention studies	Study 1: Adults of both sexes aged 18–35 years (*n* = 22) Study 2: Female adults aged 18–35 years (*n* = 10)	In Study 1, participants consume Diet Rite Cola™ or unsweetened sparkling water three times daily. In Study 2, all participants follow a single-arm pre/post design and consume Diet Pepsi™ three times daily. Both beverages contain sucralose and acesulfame-K. The estimated sucralose intake is ~204 mg/day for 1 week in Study 1 and for 8 weeks in Study 2.	~3.4 mg/kg/day Below FDA ADI (0.68×)	16S rRNA gene sequencing (V2–V3)	Study 1, sucralose-containing beverage arm vs. control: ↑ *Parabacteroides timonensis*, *Shigella boydii*, *Enterocloster asparagiformis*, *Halomonas heilongjiangensis*, *Bacteroides clarus*, *Paraprevotella xylaniphila*, *Bacteroides kribbi*, *Faecalibacillus faecis*, *Neglecta timonensis*, bacterium LF-3, *Alistipes onderdonkii*, *Parabacteroides johnsonii*, *Subdoligranulum variabile*, *Bacteroides thetaiotaomicron*, and *Bilophila wadsworthia* Study 2, pre-treatment vs. post-treatment: ↑ Firmicutes and Gammaproteobacteria	No other significant changes were reported.	[32]
Parallel, double-blind, placebo-controlled study	Healthy men aged 18–50 years (*n* = 34)	Participants consume 260 mg of sucralose (*n* = 17) or placebo (*n* = 17) three times daily for 1 week, corresponding to a total daily sucralose intake of 780 mg. Sucralose capsules contain sucralose plus 70 mg calcium carbonate; placebo capsules contain calcium carbonate only.	~13 mg/kg/day Above FDA ADI (2.6×)	16S rRNA gene sequencing (V3–V4)	Sucralose group vs. placebo group: ↓ Bacteroidetes	Participants in the sucralose group show increased insulin levels.	[57]

Abbreviations: ADI, acceptable daily intake; EDI, estimated daily intake; FDA, U.S. Food and Drug Administration; NNS, non-nutritive sweetener; T2DM, type 2 diabetes mellitus. * The reported results correspond to sucralose exposure and do not account for the independent effects of alternative NNS when these were evaluated in the same study. Results obtained from beverages or formulations containing sucralose in combination with other sweeteners should not be interpreted as equivalent to those obtained with pure sucralose alone. ** Estimated daily intake (EDI) values were calculated based on total daily sucralose consumption and standardized to a body weight of 60 kg. These estimates are provided to facilitate comparison with the FDA acceptable daily intake (ADI) for sucralose (5 mg/kg/day). *** When indicated, the reported *n* corresponds only to the subset of participants included in microbiota analysis rather than to the total study population. ↑ indicates increased abundance or levels relative to the corresponding comparison group. ↓ indicates decreased abundance or levels relative to the corresponding comparison group.

## Data Availability

The original contributions presented in this study are included in the article. Further inquiries can be directed to the corresponding author.

## Abbreviations

The following abbreviations are used in this manuscript:

ADI	Acceptable Daily Intake
AhR	Aryl Hydrocarbon Receptor
AOM	Azoxymethane
BMI	Body Mass Index
CAC	Colitis-Associated Cancer
CA	Cholic Acid
CA/CDCA	Cholic Acid/Chenodeoxycholic Acid
CDCA	Chenodeoxycholic Acid
CRC	Colorectal Cancer
DAI	Disease Activity Index
DCA	Deoxycholic Acid
DC	Dendritic Cell
DSS	Dextran Sodium Sulfate
EFSA	European Food Safety Authority
ER	Endoplasmic Reticulum
ETBF	Enterotoxigenic Bacteroides fragilis
FDA	Food and Drug Administration
FMT	Fecal Microbiota Transplantation
FXR	Farnesoid X Receptor
GES	Gene Expression Signature
HFD	High-Fat Diet
IBD	Inflammatory Bowel Disease
ICI	Immune Checkpoint Inhibitors
IFN-γ	Interferon Gamma
IL	Interleukin
IAA	Indole Acetic Acid
IPA	Indole-3-Propionic Acid
LCA	Lithocholic Acid
LPS	Lipopolysaccharide
MDA	Malondialdehyde
MHC	Major Histocompatibility Complex
MSI-H	Microsatellite Instability-High
MyD88	Myeloid Differentiation Primary Response Protein 88
NAFLD	Non-Alcoholic Fatty Liver Disease
NF-κB	Nuclear Factor Kappa B
NLRP3	NOD-Like Receptor Family Pyrin Domain Containing 3
NNS	Non-Nutritive Sweeteners
NSCLC	Non-Small-Cell Lung Cancer
NO	Nitric Oxide
NR	Non-Responder
OGTT	Oral Glucose Tolerance Test
ORR	Overall Response Rate
OSU	Operational Subspecies Unit
PFS	Progression-Free Survival
PLCγ	Phospholipase C Gamma
PD-1	Programmed Cell Death Protein 1
ROS	Reactive Oxygen Species
RNS	Reactive Nitrogen Species
R	Responder
SCFAs	Short-Chain Fatty Acids
SFA	Saturated Fatty Acids
SLIDE	Significant Latent Factor Interaction Discovery and Exploration
SAMP	Senescence-Accelerated Mouse Prone
STAT3	Signal Transducer and Activator of Transcription 3
TCR	T-Cell Receptor
TEER	Transepithelial Electrical Resistance
TGF-β	Transforming Growth Factor Beta
T2DM	Type 2 Diabetes Mellitus
TMB	Tumor Mutational Burden
TLR	Toll-Like Receptor
TLS	Tertiary Lymphoid Structures
TNF-α	Tumor Necrosis Factor Alpha
VEGF	Vascular Endothelial Growth Factor

## Appendix A

**Table A1:** Distribution of studies included in the narrative review according to evidence category Gallery.

Category	Number	Cite
Animal Models	17	[27,28,34,35,36,37,39,40,41,42,44,47,65,76,77,78,81]
Human Studies	9	[30,31,32,55,56,57,66,67,84]
In Vitro/Basic Science	4	[45,46,75,82]
Mixed Studies (Translational)	5	[62,64,80,83,87]
TOTAL	35	
